# Supplementary material for: Brain responses and approach bias to social alcohol cues and their association with drinking in a social setting in young adult males
Source: Eur J Neurosci. 2019 Oct 3;51(6):1491–503. doi: 10.1111/ejn.14574 (PMC7155040; doi:10.1111/ejn.14574)
Supplement: Supplementary file 1 [file EJN-51-1491-s001.pdf]

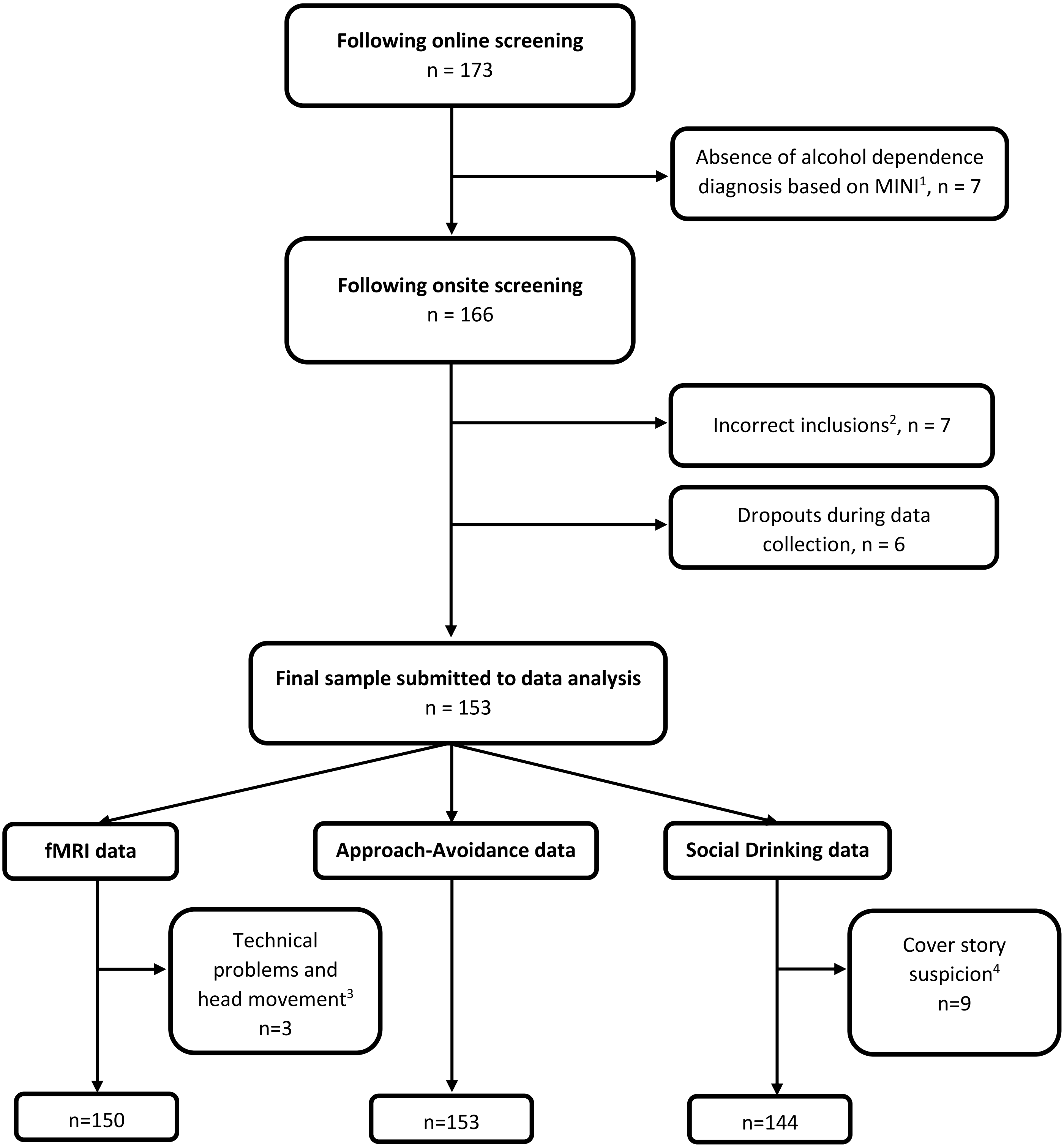

Figure S1: Flow chart of the entire data collection

[1]*In the context of the larger project, a severe group of "dependent drinkers" was defined based on AUDIT scores, weekly drinking scores and DSM criteria for alcohol dependence using a MINI interview. Some participants who showed signs of problematic and heavy alcohol use during online screening did not meet the criteria for alcohol dependence following the onsite screening. We decided to exclude these participants, as we deemed that this inconsistency between self-report and DSM-based measures made their categorization uncertain.*

[2] *Incorrect inclusions did not meet the combined requirement for the AUDIT and weekly drink score to be included in the "light drinkers" or " at-risk drinkers" group, also in the context of the larger project. The data from these 7 participants were discarded before performing any data analysis.*

[3] *For one participant, data from the fMRI session was missing, because we were unable to correct for his impaired vision. For another two participants, the fMRI data were discarded because they did not pay attention during the SACE-task as they did not respond to the control pictures using a button-press.*

[4]*Nine individuals appeared to be aware of the true purpose of the Bar-Lab sessions as they reported on 'social influence' as an aim of the study.*

Table S1: Overview of all collected data in the study

WHEN	WHAT	HOW
**SCREENING**		
	Alcohol use disorder	AUDIT
	Drinks last week	TLFB
	Gender	Male/female question
	Age	Open question
**MINI INTERVIEW (for the dependent group only)**		
	Alcohol dependence diagnosis	MINI
	Other substance use information	MINI
**BEHAVIOURAL SESSION 1**		
	Education	Multiple choice
	Age of first alcohol consumption	Open question
	Frequency of drinks in the last 4 weeks	Multiple choice
	Binge drinking episodes in last 4 weeks	Multiple choice
	Location of alcohol consumption	Multiple choice
	Drinking motives	DMQ-R
	Ever drank alcohol	Yes/No question
	Number of hours since last drink	Open question
	Ever smoked in life	Yes/No question
	Current smoker	Yes/No question
	Number of hours since last cigarette	Open question
	Smoking severity	FTND
	Ever used drugs (sleeping pills/cannabis/cocaine/ecstacy/amphetamine/hallucinogens/opiates), if yes how many times	Open question
	Impulsivity	BIS11
	State-trait Anxiety	STAI
	Depression	BDI
	Drinking Urge	DAQ
	Imitation of alcohol use	Number of drinks consumed in Bar-lab **(current paper)**
	Confederate liking ratings	1-9 scales
	Anxiety before/during/after session	Multiple choice
	Suspicion checks about study goals	Open questions
**BEHAVIOURAL SESSION 2**		
	Number of hours since last drink	Open question
	Number of hours since last cigarette	Open question
	Delay Discounting	Delay discounting task
	Drinking Urge	DAQ
	Imitation of alcohol use	Number of drinks consumed in Bar-lab **(current paper)**
	Confederate liking ratings	1-9 scales
	Anxiety before/during/after session	Multiple choice
	Suspicion checks about study goals	Open questions

Table S1: Overview of all collected data in the study (continued)

**FMRI SESSION**		
**WHEN**	**WHAT**	**HOW**
	Outside the MRI scanner	
	Number of hours since last drink	Open question
	Number of hours since last cigarette	Open question
	Height/Weight	Open question
	Drinking Urge	DAQ
	Drinking Self-Efficacy	DRSEQ
	Approach/Avoidance of (social) alcohol pictures	Stimulus-Response Compatibility task **(current paper)**
	Inside the MRI scanner	
	Brain responses to social alcohol cues	Social Alcohol cue Reactivity task **(current paper)**
	Brain responses to anticipating and receiving beer	Beer Incentive Delay task
**FOLLOW UP BASELINE**		
	Alcohol use disorder	AUDIT
	Average number of drinks for each day of the week	Weekly drinking
	Drinking motives	DMQ-R
**FOLLOW UP WITH ECOLOGICAL MOMENTARY ASSESSMENT (14 DAYS)**		
	Number of (non)alcohol units consumed day before	Open question
	Location of (non)alcohol consumption day before	Multiple choice
	People with whom (non)alcohol was consumed with day before	Multiple choice

Table S2: Whole brain activations for the different contrasts in the SACE task

Brain area	Hemisphere	Cluster size	MNI coordinates peak voxel			T-value	p-value
			x	y	Z		
Interaction ((SA>SS)-(NA>NS))							
**Superior Temporal Sulcus**	**R**	**104**	**54**	**-4**	**-11**	**4.75**	**0.005**
Superior Temporal Sulcus	R		51	5	-14	4.17	
Superior Temporal Sulcus	R		48	-10	-8	4.02	
**Superior Temporal Sulcus**	**L**	**103**	**-51**	**8**	**-8**	**4.48**	**0.006**
Superior Temporal Sulcus	L		-60	5	19	3.80	
Superior Temporal Sulcus	L		-54	-1	-20	3.45	
**Inferior Parietal Lobe**	**L**	**66**	**-60**	**-28**	**25**	**4.16**	**0.036**
Alcohol > Soda ((SA+NA)-(SS+NS))							
**Visual Cortex**	**L**	**2359**	**-15**	**-88**	**-11**	**16.37**	**0.000**
Visual Cortex	R		18	-94	1	13.89	
Visual Cortex	L		-18	-100	7	13.60	
**Superior Frontal Gyrus**	**L**	**430**	**-3**	**35**	**-5**	**5.70**	**0.000**
Anterior Cingulate Cortex	L		-3	17	-6	4.84	
Anterior Cingulate Cortex	L		-12	41	-11	4.43	
**ventral medial Prefrontal Cortex/ Subgenual Frontal Cortex**	**L/R**	**424**	**0**	**11**	**55**	**5.66**	**0.000**
ventral medial Prefrontal Cortex/ Subgenual Frontal Cortex	L/R		0	23	31	5.55	
Superior Frontal Gyrus	L		-3	17	40	5.42	

Table S2: Whole brain activations for the different contrasts in the SACE task

**Superior parietal gyrus**	**L**	**307**	**-15**	**-52**	**10**	**4.89**	**0.000**

Superior parietal gyrus	L		-6	-61	13	4.73	
Superior parietal gyrus	R		18	-52	13	4.58	
Social > Non-Social ((SA+SS)-(NA+NS))							
**Cuneus**	**R**	**14291**	**12**	**-88**	**1**	**26.22**	**0.000**
Lingual Gyrus	L		-9	-88	-5	24.72	
Lingual Gyrus	R		12	-85	-8	24.30	
**Middle frontal gyrus**	**R**	**908**	**45**	**14**	**31**	**11.93**	**0.000**
Inferior Frontal Gyrus	R		57	29	19	11.31	
Inferior frontal gyrus	R		48	23	22	10.78	
**Ventral medial Prefontal Cortex/Posterior orbital gyrus**	**R**	**69**	**33**	**35**	**-14**	**8.55**	**0.047**
ventral medial Prefrontal Cortex /Posterior orbital gyrus	R		30	29	-2	4.52	
**Superior Frontal Gyrus**	**L**	**109**	**-6**	**14**	**49**	**6.19**	**0.008**
Superior Frontal Gyrus	R		6	14	49	5.97	

Note: Reported coordinates correspond to the three highest peak voxels more than 8 mm apart in each cluster. P-values are cluster level FWE-corrected.

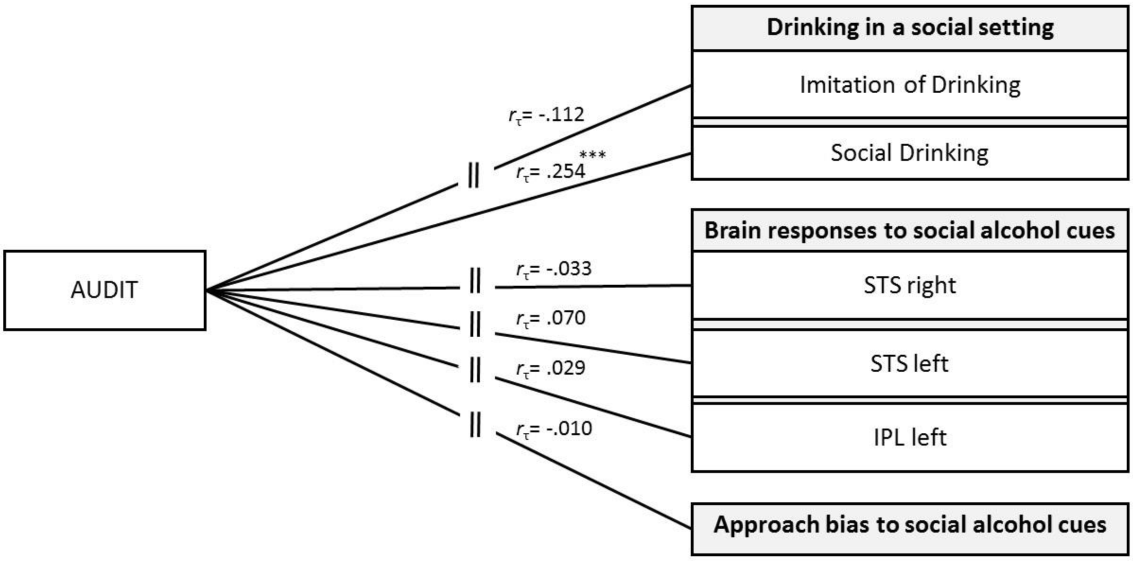

Figure S2: Exploratory correlational analyses investigating the association between AUDIT and main outcomes measures.

Notes: Abbreviations: AUDIT = Alcohol Use Disorder Identification Test; STS = Superior Temporal Sulcus; IPL = Inferior Partietal Lobe. AUDIT and imitation of drinking (r_τ= -.112, p= .076 | BF_{01}= 1.276), AUDIT and social drinking (r_τ= .254, p<.001 | BF_{01} <0.001), AUDIT and brain responses to social alcohol cues: STS right: r_τ=-.033, p=.566 | BF_{01}= 7.654; STS left: r_τ=.070, p=.232 | BF_{01}= 4.318; IPL left: r_τ=.029, p=.613 | BF_{01}= 7.951, AUDIT and approach bias towards social alcohol cues (r_τ=-.010, p=.863 | BF_{01}= 9.036).

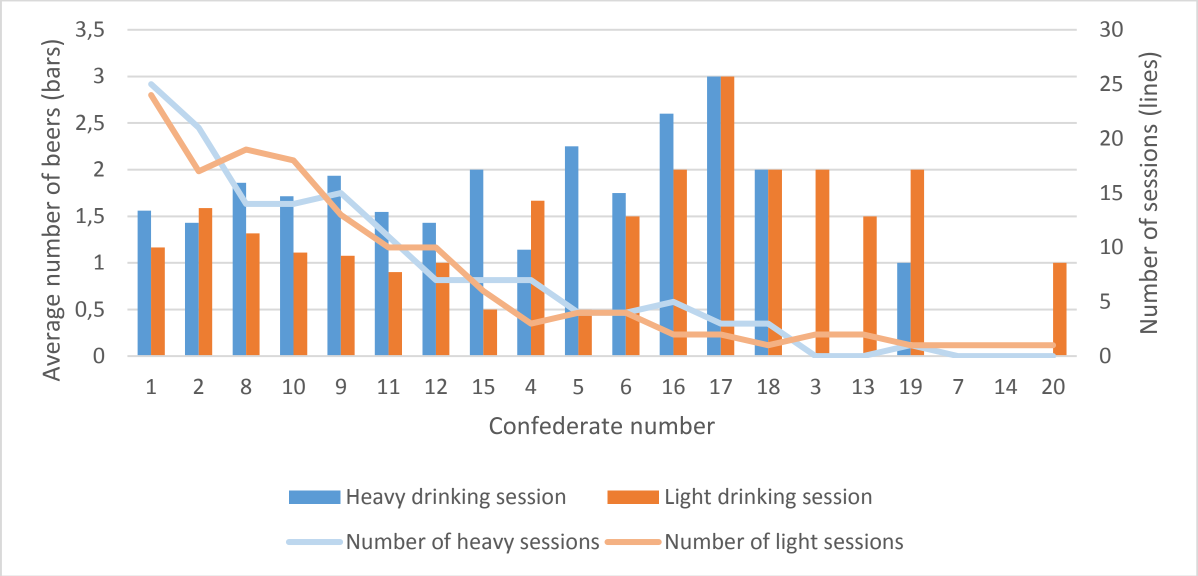

Figure S3a: For each confederate is plotted 1) the average number of beers consumed by the participants in the company of this particular confederate (bars), and 2) the number of “light” and “heavy” drinking sessions that this confederate participated in (lines)

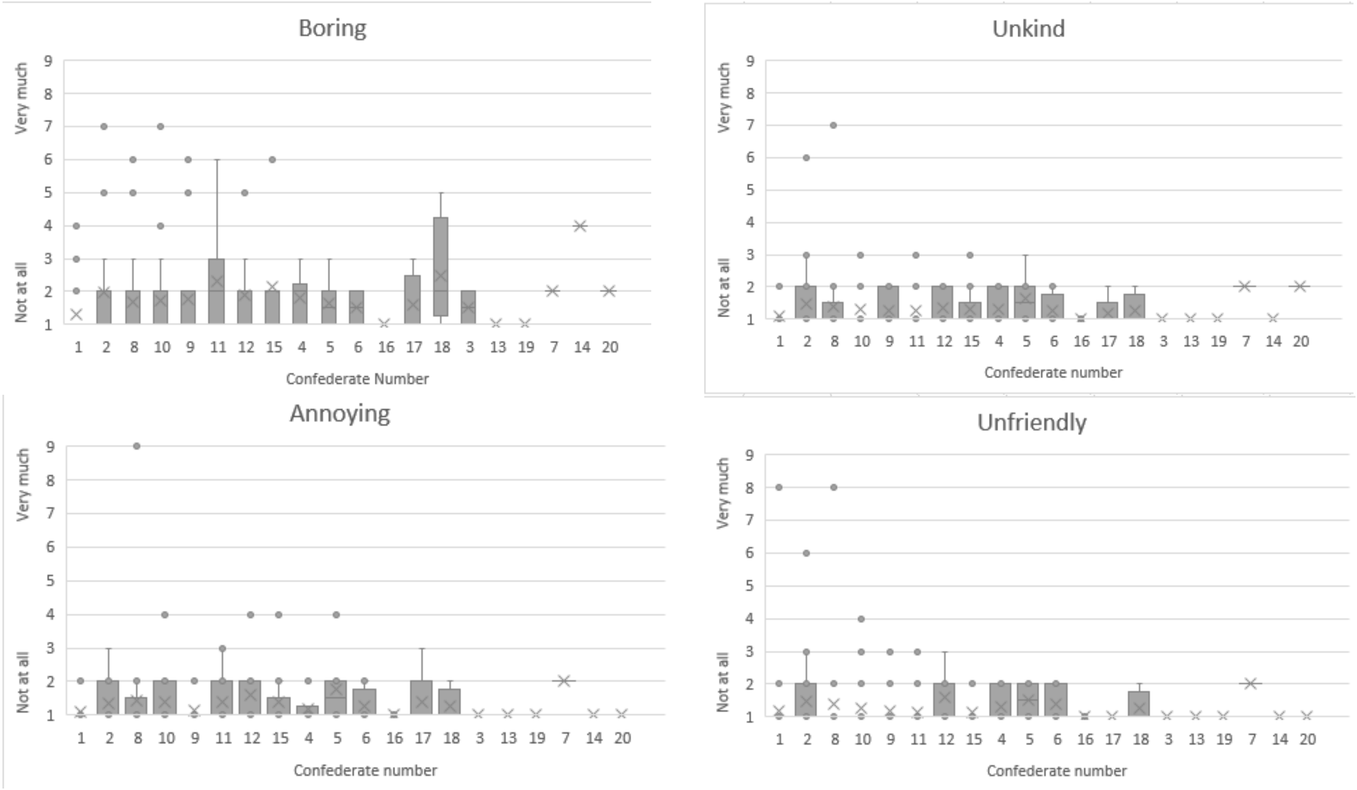

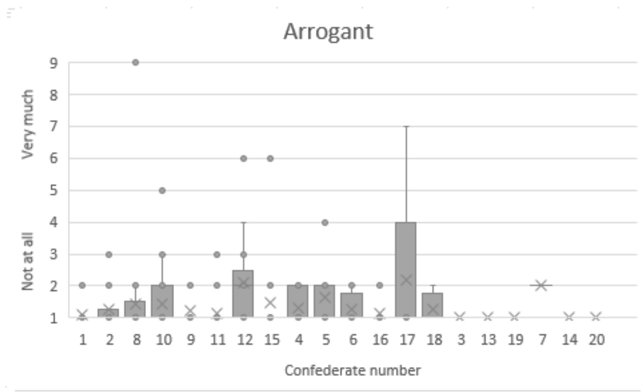

Figure S3b: Boxplots for evaluation of confederates

Notes: Confederates are ordered based on the number of sessions participated, with more session on the left. Dots are outliers and crosses are means.

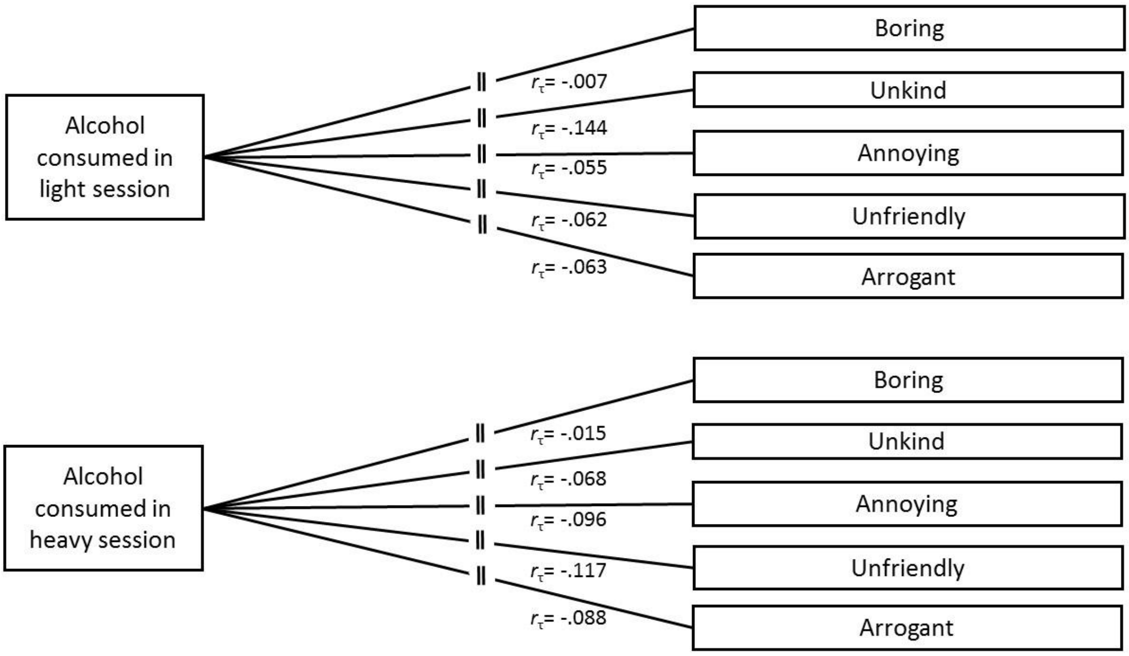

Figure S3c: Correlation between number of drinks consumed by participant and evaluation scores of confederates

Notes: In the light drinking session: Boring: r_{τ}=-.007, p=.926 | BF_{01} = 8.985; Unkind: r_{τ}=-.144, p=.064 | BF_{01} = 0.392; Annoying: r_{τ}= -.055, p=.479 | BF_{01} = 5.734; Unfriendly: r_{τ}=-.062, p=.423 | BF_{01} = 5.049; Arrogant: r_{τ}=-.063, p=.415 | BF_{01} = 4.870.
In the heavy drinking session: Boring: r_{τ}=-.015, p=.839 | BF_{01} = 8.783 ; Unkind: r_{τ}=-.068, p=.366 | BF_{01} = 4.421; Annoying: r_{τ}= -.096, p=.208 | BF_{01} = 2.231; Unfriendly: r_{τ}=-.117, p=.123 | BF_{01} = 1.104; Arrogant: r_{τ}=-.088, p=.243| BF_{01} = 2.736.
