# Supplementary material for: Brain responses and approach bias to social alcohol cues and their association with drinking in a social setting in young adult males
Source: Eur J Neurosci. 2019 Oct 3;51(6):1491–503. doi: 10.1111/ejn.14574 (PMC7155040; doi:10.1111/ejn.14574)
Supplement: Supplementary file 2 [file EJN-51-1491-s002.docx]

**FIGURE LEGENDS**

Figure 1: Bar-lab; overview and close-up on the bar. The participants and confederates were sitting on the barstools.

Figure 2: Brain responses during the Social Alcohol Cue Exposure task (A) Whole brain analysis of cue reactivity to social alcohol pictures, i.e. interaction contrast [(Social Alcohol (SA)> Social Soda (SS))-(Non-social Alcohol (NA) > Non-Social Soda (NS))]. Boxplots -reported for illustrative purposes- show the percent signal change (PSC) in the three functional clusters that show a significant interaction effect in the whole brain analysis. (B) Whole brain analysis of cue reactivity to alcohol pictures and social pictures, i.e. main effect contrasts [(Social Alcohol + Non-Social Alcohol)-(Non-Social Soda + Social Soda)] in red, and [(Social Alcohol + Social Soda) – (Non-Social Alcohol + Non-Social Soda)] in blue. Display threshold for panels A and B: voxel-level uncorrected p<.001 combined with cluster-level FWE corrected p<.05. Social= Social Alcohol-Social Soda, Non-Social= Non-Social Alcohol- Non-Social Soda, ACC = Anterior Cingulate Cortex, vmPFC = ventral medial Prefrontal Cortex.

Figure 3: Boxplots of approach bias scores (reaction time for Avoid – Approach condition in ms) for the 4 main conditions. SA=Social Alcohol, SS= Social Soda, NA= Non-Social Alcohol, NS= Non-Social Soda. There is a significant approach bias in all conditions, as well as a main effect of Drink (p=.001), with a stronger approach bias towards Alcohol compared with Soda pictures.

Figure 4: Overview of the results. Broken lines reflect no significant associations between the variables. STS = Superior Temporal Sulcus, IPL = Inferior Parietal Lobe
